# *Leishmania*-Induced IRAK-1 Inactivation Is Mediated by SHP-1 Interacting with an Evolutionarily Conserved KTIM Motif

**DOI:** 10.1371/journal.pntd.0000305

**Published:** 2008-12-23

**Authors:** Issa Abu-Dayyeh, Marina Tiemi Shio, Shintaro Sato, Shizuo Akira, Benoit Cousineau, Martin Olivier

**Affiliations:** 1 Department of Microbiology and Immunology, McGill University, Montréal, Québec, Canada; 2 Centre for the Study of Host Resistance, The Research Institute of the McGill University Health Centre, Montréal, Québec, Canada; 3 Research Institute of Microbial Diseases, Osaka University, Osaka, Japan; The George Washington University, United States of America

## Abstract

Parasites of the *Leishmania* genus can rapidly alter several macrophage (MØ) signalling pathways in order to tame down the innate immune response and inflammation, therefore favouring their survival and propagation within their mammalian host. Having recently reported that *Leishmania* and bacterial LPS generate a significantly stronger inflammatory response in animals and phagocytes functionally deficient for the Src homology 2 domain-containing protein tyrosine phosphatase (SHP-1), we hypothesized that *Leishmania* could exploit SHP-1 to inactivate key kinases involved in Toll-like receptor (TLR) signalling and innate immunity such as IL-1 receptor-associated kinase 1 (IRAK-1). Here we show that upon infection, SHP-1 rapidly binds to IRAK-1, completely inactivating its intrinsic kinase activity and any further LPS-mediated activation as well as MØ functions. We also demonstrate that the SHP-1/IRAK-1 interaction occurs via an evolutionarily conserved ITIM-like motif found in the kinase domain of IRAK-1, which we named KTIM (Kinase Tyrosyl-based Inhibitory Motif). This regulatory motif appeared in early vertebrates and is not found in any other IRAK family member. Our study additionally reveals that several other kinases (e.g. Erk1/2, IKKα/β) involved in downstream TLR signalling also bear KTIMs in their kinase domains and interact with SHP-1. We thus provide the first demonstration that a pathogen can exploit a host protein tyrosine phosphatase, namely SHP-1, to directly inactivate IRAK-1 through a generally conserved KTIM motif.

## Author Summary

*Leishmania* developed several methods to seize control of macrophage signalling pathways in an effort to inactivate their killing abilities. One effective method utilized by the parasite is the activation of host protein tyrosine phosphatases, specifically SHP-1. This increased phosphatase activity contributes to the inactivation of signalling molecules involved in critical macrophage functions such as NO and cytokine production. Interestingly, the absence of SHP-1 results in stronger macrophage inflammatory responses to a bacterial cell wall component known as LPS, a molecule detected by macrophages through Toll-like receptors (TLRs). This observation suggested a role for SHP-1 in the regulation of TLR signalling. Our study reveals that upon *Leishmania* infection, SHP-1 is able to rapidly bind to and inactivate a critical kinase (IRAK-1) in this pathway. This regulatory binding was shown to be mediated by an evolutionarily conserved motif identified in the kinase. This motif was also present in other kinases involved in Toll signalling and therefore could represent a regulatory mechanism of relevance to many kinases. This work not only reports a unique mechanism by which *Leishmania* can avoid harmful TLR signalling, but also provides a platform on which extensive investigation on host evasion mechanisms and regulation of cellular kinases can be gained.

## Introduction

Innate inflammatory responses play a critical role in controlling pathogens [1]. However, protozoan parasites such as *Leishmania* evolved strategies to avoid phagocyte activation by seizing control of key signalling pathways, therefore favouring their invasion and survival within the host cell [2]. We recently reported that the protein tyrosine phosphatase (PTP) SHP-1 plays a pivotal role in taming down phagocyte-mediated inflammatory responses [3]. For instance, we showed that in the absence of SHP-1, several pro-inflammatory cytokines (e.g. IL-1β, IL-6, TNFα) and chemokines, as well as inflammatory neutrophil recruitment were all exacerbated by *Leishmania* infection [3]. Of interest, we also found that LPS mediates an excessive inflammatory response in the absence of SHP-1, therefore suggesting that SHP-1 could exert its negative regulatory action via Toll like receptor (TLR) signalling.

As SHP-1 can interact with various members of the JAK and MAP kinase families in physiological, immune response, and infection contexts [2],[3], we explored the possibility that the capacity of *Leishmania* to block the macrophage (MØ) inflammatory response could result from rapid IRAK-1 kinase inactivation through SHP-1 action. This hypothesis is further reinforced by the fact that several LPS-mediated MØ functions (e.g. TNFα, NO, IL-12), critical for the containment of pathogens and adaptive immune response development, are inhibited upon *Leishmania* infection [2],[4],[5].

Whereas invertebrates depend mainly on the evolutionarily conserved innate immune system to fight off pathogens, vertebrates have developed a sophisticated adaptive immune system, hence the need to regulate the innate immune response. The TLR family has been shown to play a key role in triggering innate immunity as well as the subsequent induction of adaptive immune responses in vertebrates [6]. Our previous findings reporting augmented *Leishmania*- and LPS-induced innate inflammatory response in the absence of SHP-1 (PTPN6) [3], and the several reports that key transcription factors (NF-κB and AP-1) related to TLR signalling were strongly activated in the absence of SHP-1 [7]–[9], suggested the importance of SHP-1 in the negative regulation of TLR signalling and its subsequent inflammatory response in vertebrates. Of interest, a mutation in the *PTPN6* gene coding for SHP-1 in humans has been recently linked to Sezary syndrome [10], a T-cell cutaneous lymphoma arising from chronic inflammatory state.

From these observations, and given the fact that IRAK-1 serves as a crucial kinase in all MyD88-dependent pathways leading to the activation of innate inflammatory responses, we hypothesised that SHP-1 is a critical player in the negative regulation of this kinase that can be exploited by *Leishmania*. For instance, until recently there was no indication that SHP-1 could interact with IRAK-1. However, a recent study by Cao's laboratory [11] provided strong evidence that SHP-1 can interact with IRAK-1.

Here, we provide evidence that SHP-1 negatively regulates IRAK-1 intrinsic kinase activity in its resting state and upon *Leishmania* infection through binding to an evolutionarily conserved ITIM-like motif located within IRAK-1's kinase domain. In addition, it is important to stress that this is the first mention of this motif to be found within a kinase, as to date it has only been found within the intracytoplasmic portion of immunoglobin (Ig)-like receptors. Of interest, we also discovered that this ITIM-like motif was present in several other kinases. Finally, our study also provides evidence from *in silico* sequence analyses that both IRAK-1 and SHP-1 evolutionarily emerged in vertebrates concomitantly with the development of a better-controlled innate immune response. Therefore the appearance of this key interaction in early vertebrates may have also contributed to the development of the more complex adaptive immune response.

## Materials and Methods

### Cell culture and reagents

The immortalized me-3 (SHP-1^−/−^) and LM-1 (WT) bone marrow-derived MØs (BMDMs) were generated from motheaten mice (*Ptpn6^me/me^*; C3HeBFeJ *me*/*me*) and their respective wild-type littermates (C3HeBFeJ *me*/+) as described [7]. The immortalized B10R BMDMs were derived from B10A.Bcg^r^ mice [12]. L929 cells used for the TNF bioassays were grown in RPMI-1640 medium (5% FBS). MØ-activating lipopeptide-2 (MALP-2) and lipopolysaccharide (LPS) from *E.coli* were purchased from Alexis Biochemicals, San Diego, CA. Flagellin and CpG DNA were purchased from Invivogen, San Diego, CA. IRAK1/4 inhibitor (N-(2-Morpholinylethyl)-2-(3-nitrobenzoylamido)-benzimidazole) was purchased from Calbiochem, La Jolla, CA.

### *In vitro* infection

*L. donovani infantum, L. mexicana* (MNYC/BZ/62/M379), *L. major* Friedlin strain (MHOM/JL/80/Friedlin), and *L. tarentolae* strain TAR II promastigotes were kept in SDM medium (10% FBS), and stationary phase parasites were used to infect cells in a parasite to MØ ratio of 20∶1. Non-internalized parasites were removed by washing the plates with phosphate-buffered saline (PBS), after which MØs were collected for subsequent experiments.

### Western blot analysis

Western blotting was performed as previously described [13]. Proteins were detected using antibodies directed against IRAK-1 (generated in the laboratory of Dr. Akira), SHP-1 and phospho-tyrosine (clone 4G10) (Upstate, Charlottesville, VA), and actin (Sigma-Aldrich, ON, Canada). Proteins were detected using an anti-rabbit or anti-mouse horseradish peroxidase (HRP)-conjugated antibody (Amersham, QC, Canada) and visualized using ECL western blotting detection system (Amersham).

### IRAK-1/IRAK-4 kinase assay

6×10^6^ MØs were lysed in cold lysis buffer (20mM Tris (pH 7.5), 1mM EDTA, 150mM NaCl, 1% Igepal, 10mM β-glycerophosphate, 1mM sodium orthovanadate, 25 µg/ml aprotinin and 25 µg/ml leupeptin). Lysates were precleared with protein A/G agarose beads (Santa Cruz, CA). Samples were then centrifuged (13,000× g, 10 min) and supernatants kept. IRAK-1 or IRAK-4 antibody and protein A/G agarose beads were added to the supernatant and samples were incubated O/N at 4°C. Beads were spun down and washed with the lysis buffer described above, followed by washes with the kinase assay buffer (20mM HEPES pH 7.5, 20mM MgCl_2_, 3mM MnCl_2_, and 10mM β-glycerophosphate). Kinase assay buffer (20 µl) containing 10 µCi of γ-^32^P (Amersham) was then added to the beads and samples incubated (30 min, 30°C). The reaction was stopped by the addition of 4× sample loading buffer (12.5% Tris-HCl (pH 6.8), 10% glycerol, 10% SDS, 5% β-mercaptoethanol, 0.05% bromophenol blue). Samples were boiled and ran on SDS-PAGE. Bands were detected using X-ray Kodak films (Amersham) or by image analyzer (BioRad, Canada).

### In gel PTP assay

For immunoprecipitation samples, 6×10^6^ MØs were lysed as described previously for the IRAK-1 kinase assay without the addition of sodium orthovanadate to the lysis buffer. Cell lysate controls (25 µg) were obtained using a PTP lysis buffer (50mM Tris (pH 7.0), 0.1mM EDTA, 0.1mM EGTA, 0.1% β-mercaptoethanol, 1% Igepal, 25 µg/ml aprotinin and 25 µg/ml leupeptin). Samples were loaded on a gel containing a γ-^32^P-labelled poly(Glu4Tyr) peptide (Sigma-Aldrich) and the SHP-1 band was observed by in gel PTP assay as previously described [14].

### Co-immunoprecipitation

Samples were lysed in the western blot lysis buffer (no sodium orthovanadate was added when immunoprecipitating SHP-1) and immunoprecipitated using protein A/G agarose beads (Santa Cruz) and 4 µg of the IRAK-1, SHP-1 antibody, or anti-rat antibody (Sigma-Aldrich) for non-specific binding. Beads were spun down and washed three times with lysis buffer. Beads were resuspended in the 4× western sample loading buffer previously described and boiled supernatants were loaded on SDS-PAGE and western blot analysis was performed as described above.

### GST pull-down assay

Wildtype mouse IRAK-1 gene and the IRAK-1 genes of the different KTIM mutants (all in PCDNA3 vectors) were *in vitro* translated using the Promega TNT Quick Coupled Transcription/Translation kit (Fisher Scientific, ON, Canada) using 20 µCi ^35^S (Amersham). The active or the trapping mutant of GST SHP-1 was produced in BL21 bacteria. Bacterial lysates were extracted using the BugBuster Protein Extraction Reagent (VWR CANLAB, ON, Canada), and the GST protein (5 µg) was pulled down from bacterial lysates using glutathione sepharose beads (30 µl) (Amersham). The active/trapping mutant of GST-SHP-1 bound to glutathione beads was left to interact with immunoprecipitates (IPs) or *in vitro* translated IRAK-1 protein in a PTP reaction buffer (50mM Hepes (pH 7.5), 0.1% β-mercaptoethanol) for 1 h at RT. When in vitro translation of IRAK-1 was performed, GST-SHP-1 was allowed to interact with IRAK-1 in a 5∶1 ratio. Beads were then spun down, washed 3× with the PTP lysis buffer, then resuspended in 4× sample loading buffer (20 µl), boiled, and loaded on SDS-PAGE. IRAK-1 bands were revealed by exposing to X-ray film (Amersham).

### Alkali-resistance phosphoprotein assay

Kinase assays were run on SDS-PAGE as described above, pre-treatment image is taken by exposing the gel to a phospho-imager screen. Next, gels were fixed overnight at RT in a 10% methanol/7% acetic acid solution. Gels were then soaked in a 10% glutaraldehyde solution (30 min, RT) with gentle shaking and rinsed in water prior to incubation with KOH. The alkali treatment of ^32^P-labelled IRAK-1 was performed as previously described [15].

### Generation of IRAK-1 mutants

The mouse IRAK-1 gene cloned into a PCDNA3 plasmid was mutated at different sites within the KTIM using the QuikChange site-directed mutagenesis kit (Stratagene, La Jolla, CA) as instructed by the manufacturer. The primers (all synthesized by Genome Québec, Montréal, QC, Canada) designed to create the mutants were:

For the tyrosine to phenylalanine mutation;

sense: 5′GGCTTATACTGCCTTGTTTTTGGCTTCTTGCCCAATGG3′;

anti-sense: 5′CCATTGGGCAAGAAGCCAAAAACAAGGCAGTATAAGCC3′.

For the leucine to methionine mutation;

sense: 5′GGCTTATACTGCCTTGTTTATGGCTTCATGCCCAATGG3′;

anti-sense: 5′CCATTGGGCATGAAGCCATAAACAAGGCAGTATAAGCC3′.

For the glycine to alanine, phenylalanine to tyrosine, leucine to methionine triple mutation, sequential mutagenesis was performed where the above-mentioned leucine mutation was used as the template to generate an additional glycine to alanine mutation using the primers:

sense: 5′GGCTTATACTGCCTTGTTTATGCCTTCATGCCCAATGG3′;

anti-sense: 5′CCATTGGGCATGAAGGCATAAACAAGGCAGTATAAGCC3′.

Finally, a phenylalanine to tyrosine mutation was generated using the previously described double mutation as a template using the primers:

sense: 5′GGCTTATACTGCCTTGTTTATGCCTACATGCCCAATGG3′;

anti-sense: 5′CCATTGGGCATGTAGGCATAAACAAGGCAGTATAAGCC3′.

All mutations were verified by sequencing the entire plasmid using the T7 and SP6 primers (provided by Genome Quebec, Montreal, QC, Canada) and the internal primers 5′TTCCTCCACCAAGTCAAG3′ and 5′CCTGAGGAGTACATCAAGAC3′.

### IL-12 mRNA expression analysis

RNA was extracted from MØs using TRIzol reagent (Invitrogen Canada, ON, Canada). Reverse transcription was performed using oligodT. Quantitative Real-Time PCR (qRT-PCR) was performed with a Corbett Research Rotorgene (Corbett Life Science, Sydney, Australia), using Invitrogen Platinum SYBR Green qPCR SuperMix-UDG (Invitrogen) and 0.4 µM primer in 25 µl. qPCR program is: 50°C 2 min; 95°C 3 min; (95°C 20 sec, 60°C 30 sec, 72°C 20 sec) for 40 cycles followed by a melting curve. All primers annealing temperature was 60°C. Oligo sequences are: GAPDH: 5′-CGG ATT TGG CCG TAT TGG GCG CCT-3′ and 3′- ACA TAC TCA GCA CCG GCC TCA CCC-5′; IL-12: 5′- GGA AGC ACG GCA GCA GAA TA-3′ and 3′-AAC TTG AGG GAG AAG TAG GAA TGG-5′.

### TNF bioassay

TNF bioassay was performed as previously described [16]. Briefly, TNF-sensitive L929 fibroblasts were seeded in 96-well plates in a concentration of 3.5×10^4^ cells/100 µl/well in RPMI-1640 (5% FBS) medium and incubated for 24 h until obtaining a monolayer. Supernatants from designated experiments were added to L929 cells and serially diluted in the presence of actinomycin D (2 µg/ml). After incubation (18–24 h, 37°C), the L929 monolayers were stained with crystal violet, washed with distilled water, and left to dry. Then, methanol was added to dissolve the stain and cytotoxicity was determined by measuring absorbance at 595 nm. One unit of TNF was referred to as the reciprocal of the dilution that induced 50% of L929 cell lysis.

### NO assay

NO production was evaluated by measuring the accumulation of nitrite in the culture medium by the Griess reaction, as previously described [3].

### Electrophoretic mobility shift (EMSA)

Nuclear extracts were prepared by a standard protocol, and EMSAs were performed as previously described [17]. Briefly, nuclear extracts were incubated with binding buffer containing 1.0 ng of [γ-^32^P] dATP radiolabeled double-stranded DNA oligonucleotide for 20 min at room temperature. The DNA binding consensus sequence used for NF-κB was (5′-AGTTGAGGGGACTTTCCCAGGC-3′). Sp1 consensus oligonucleotide was used as non-specific control (5′-ATTCGATCGGGGCGGGGCGAGC-3′) (Santa Cruz). DNA-protein complexes were resolved by electrophoresis in native 4% (w/v) polyacrylamide gels. The gels were then dried and autoradiographed.

### pNPP phosphatase assay

MØs were collected, lysed in the PTP lysis buffer described previously and kept on ice for 45 min. Lysates were cleared by centrifugation, and protein content was determined by Bradford reagent followed by IP. Equal amounts of IPs were incubated in a phosphatase reaction mix (50mM Hepes (pH 7.5), 0.1% β-mercaptoethanol, 10mM pNPP) overnight at 37°C. OD was then read at 405 nm.

### Sequence alignments

Sequences were obtained from the NCBI protein database. Sequence alignments used to calculate identity and similarity percentages were generated by EMBOSS local pair-wise alignment algorithms program (http://www.ebi.ac.uk/Tools/emboss/index.html). The accession numbers of the protein sequences included in the study are: human (*Homo sapiens*) IRAK-1 (P51617), chimpanzee (*Pan troglodytes*) IRAK-1 (XP_521332), dog (*Canis familiaris*) IRAK-1 (XP_549367), bull (*Bos Taurus*) IRAK-1 (Q2LGB3), mouse (*Mus musculus*) IRAK-1 (Q62406), rat (*Rattus norvegicus*) IRAK-1 (XP_001057078), tropical frog (*Xenopus tropicalis*) IRAK-1 (NP_001006713), zebrafish (*Danio rerio*) IRAK-1 (XP_697688), human IRAK-4 (Q9NWZ3), chimpanzee IRAK-4 (XP_001166114), rhesus monkey (*Macaca mulatta*) IRAK-4 (XP_001091707), dog IRAK-4 (XP_543727), bull IRAK-4 (Q1RMT8), mouse IRAK-4 (Q8R4K2), rat IRAK-4 (XP_217026), chicken (*Gallus gallus*) IRAK-4 (NP_001025909), zebrafish IRAK-4 (AAT37635), squid (*Euprymna scolopes*) IRAK-4 (AAY27972), sea urchin (*Strongylocentrotus purpuratus*) IRAK-4 (XP_784716), *Caenorhabditis elegans* IRAK-4 (NP_502587), honeybee (*Apis mellifera*) pelle-like protein (XP_624002), *Drosophila melanogaster* pelle (NP_476971), chicken IRAK-2 (NP_001025776), mouse JAK2 (Q62120), mouse JAK3 (Q62137), mouse TAK1 (Q62073), mouse Erk1 (Q63844), mouse Erk2 (P63085), mouse JNK (CAC88132), mouse p38 (P47811), mouse IKK-α (Q60680), mouse IKK-β (O88351), mouse LYN (AAH31547).

### Band quantification

All densitometric analyses were performed using the Quantity One software, Biorad Laboratories Inc. Values and standard deviations observed represent scans of three independent experiments.

### Ethical oversight

The bone marrow-derived macrophages described in this study have been previously derived from WT and SHP-1 deficient mice (see reference 7), and immortalized as cell lines. However, experiments done on the animals used in that study (reference 7) adhered to McGill University's guidelines for animal husbandry and was approved by the institutional research ethics committee.

## Results

### SHP-1 regulates IRAK-1 kinase activity by direct interaction

**Figure 1:**
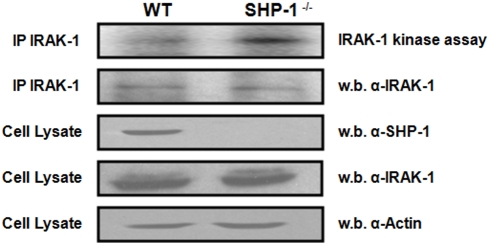
Regulation of IRAK-1 kinase activity by SHP-1. Upper Panel represents an *in vitro* kinase assay comparing the basal kinase activity of IRAK-1 in WT littermates versus *Ptpn6^me/me^* MØ (SHP-1^−/−^). A fraction of the IP was kept and subjected to a western blot as a control for equal IRAK-1 IP (2^nd^ panel from top). Cell lysates of WT and SHP-1^−/−^ MØs were blotted for SHP-1 to demonstrate the presence/absence of the SHP-1 protein (3^rd^ panel from top). The membrane was stripped and reblotted for IRAK-1 to monitor its expression level in both cell lines (4^th^ panel from top). Actin levels are shown as loading controls (bottom panel). All results are representative of at least three independent experiments.

To investigate the effect of SHP-1 on IRAK-1 kinase activity, we immunoprecipitated IRAK-1 from the lysates of SHP-1^−/−^ MØs and their wildtype (WT) counterparts and subjected the IP to an IRAK-1 kinase assay. Results indicated that IRAK-1 kinase activity in SHP-1^−/−^ cells was significantly higher compared to WT (Figure 1, top panel). The increase in IRAK-1 basal kinase activity observed in SHP-1^−/−^ cells is not due to a differential expression of IRAK-1 as supported by loading controls provided (Figure 1, lower panels).

**Figure 2:**
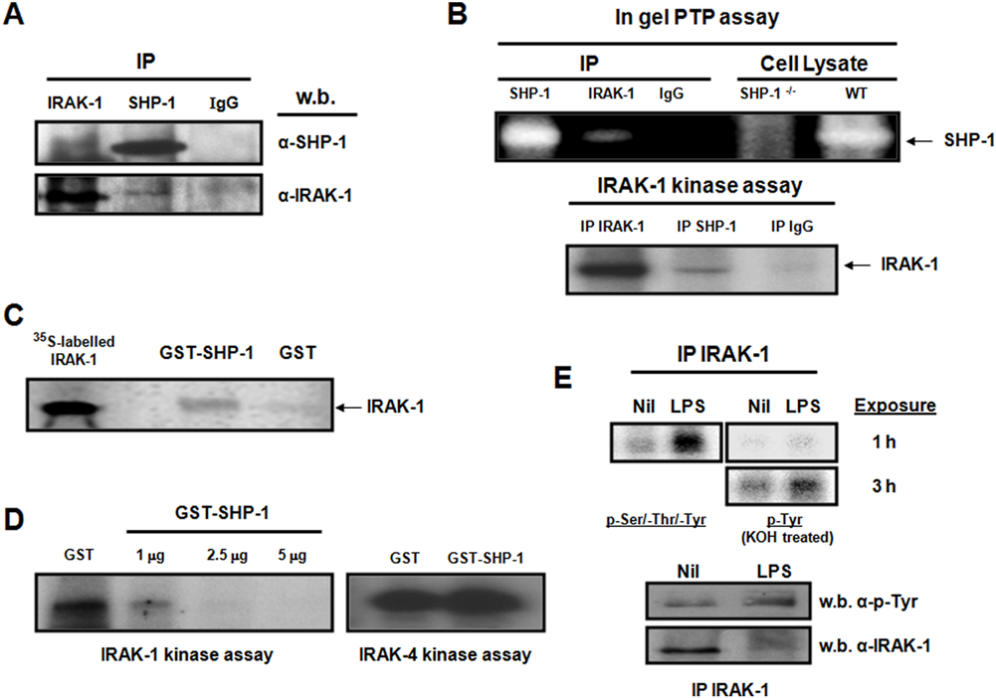
Demonstration of the IRAK-1/SHP-1 interaction. (A) Western blot analysis demonstrating the Co-IP of IRAK-1 and SHP-1. IRAK-1 and SHP-1 were immunoprecipitated then blotted against SHP-1 and IRAK-1 antibodies. Rabbit IgG anti-rat was used as a control. (B) In gel PTP activity assay of SHP-1 and IRAK-1 IPs (top blot). Rabbit IgG anti-rat was used as an IP control. Cell lysates of WT and SHP-1^−/−^ were added in the last two lanes to confirm that the signal was SHP-1. Lower blot represents IRAK-1 kinase activity in reciprocal IPs. (C) *In vitro* transcription/translation of the IRAK-1 gene was performed using radiolabelled methionine. First lane shows the IRAK-1 input. The last two lanes show methionine-labelled IRAK-1 pulled down after 1 h incubation with either a GST-SHP-1 or GST respectively. (D) Kinase assay measuring IRAK-1 activity upon its interaction with either GST alone or increasing concentrations of an active GST-SHP-1 construct (left panel). Kinase assay measuring IRAK-4 activity (right panel) upon its interaction with either GST alone or GST-SHP-1 (5 µg). (E) Kinase assay showing IRAK-1 activity at basal level and upon LPS treatment subjected to alkali treatment to evaluate tyrosine phosphorylation in IRAK-1. Tyrosyl phosphorylation was confirmed by western blot using an anti-phosphotyrosine antibody. An IP fraction was kept and blotted for IRAK-1 as a loading control.

Then, to evaluate whether the SHP-1 regulatory effect on IRAK-1's kinase activity involved their interaction, we performed immunoprecipitation assays and observed that IRAK-1 and SHP-1 co-IP (Figure 2A). Their association was further confirmed as we have detected PTP activity corresponding to SHP-1 in the IRAK-1 IP (Figure 2B, top panel), and IRAK-1 kinase activity in the IP of SHP-1 (Figure 2B, bottom panel). A secondary rat antibody was used as a negative control (Figures 2A and B). These experiments suggested the presence of IRAK-1 and SHP-1 in the same multi-protein complex. To test whether they directly interact, we *in vitro* translated IRAK-1 using radiolabelled methionine, and put the radiolabelled IRAK-1 in contact with GST-SHP-1. IRAK-1 was pulled down specifically by GST-SHP-1 and not by GST alone, showing that this interaction is direct (Figure 2C).

Next, we examined whether the binding of SHP-1 is sufficient to regulate IRAK-1 kinase activity. To do so, IRAK-1 was immunoprecipitated and put in contact with increasing concentrations of active GST-SHP-1. IRAK-1 kinase activity was inhibited in a dose-dependent manner by GST-SHP-1 and not by GST alone (Figure 2D, left panel). Interestingly, the highest dose of GST-SHP-1 used to inhibit IRAK-1 activity did not alter IRAK-4's kinase activity (Figure 2D, right panel).

The fact that the PTP-SHP-1 dephosphorylates tyrosyl residues raised the possibility that IRAK-1 is tyrosine phosphorylated. To investigate this hypothesis, alkali-resistance phosphoprotein assays were performed. Treatment of IRAK-1 kinase assay gels with KOH permits the in-gel dephosphorylation of pSer and pThr, but not pTyr allowing us to evaluate the contribution of tyrosine phosphorylation to the overall phosphorylation signal. Although IRAK-1 is known to be phosphorylated on Ser/Thr residues [18], our results represent the first demonstration that IRAK-1 is also tyrosine phosphorylated in the resting state, and that LPS increases IRAK-1 tyrosyl phosphorylation by 46±15% SD (Figure 2E, upper panels). This finding was further confirmed by western blot using the 4G10 pTyr-specific antibody (Figure 2E, lower two panels).

### SHP-1 binds to the kinase domain of IRAK-1 via an ITIM-like motif

**Figure 3:**
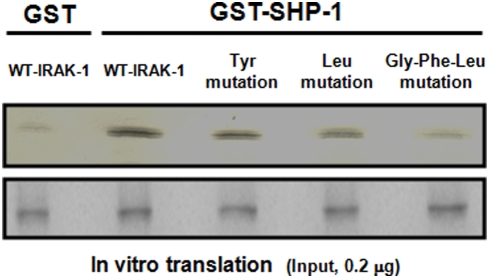
Mutation of IRAK-1's KTIM abrogates its ability to bind SHP-1. Top panel represents methionine-labelled WT-IRAK-1 as well as the different IRAK-1 mutants *in vitro* translated and put in contact with 5 µg of either GST alone or a trapping GST-SHP-1 construct. Bottom panel represents equal fractions of the *in vitro* translated products ran on an SDS gel to show equal input. All results are representative of at least three independent experiments.

At the view of our observations, we screened the mouse IRAK-1 sequence for possible SHP-1 binding sites. We discovered that IRAK-1 contains an ITIM-like motif (_286_ **L**V**Y**GF**L**_291_) located in its kinase domain (Figure S1). This motif was found to be absent in all the other IRAK family members since the last residue is a methionine instead of a leucine (Figure S2). To determine the involvement of this ITIM-like motif in the SHP-1/IRAK-1 binding, we used the full-length IRAK-1 sequence to introduce site-specific mutations within the motif followed by *in vitro* binding assays (Figure 3). Firstly, a Y288F mutation slightly decreased SHP-1 binding suggesting that possible phosphorylation of the motif's central tyrosine may increase binding affinity but is not absolutely necessary for the binding to occur. Secondly, an L291M mutation, which renders the site no more ITIM-like, significantly decreased SHP-1 binding. Thirdly, the G289A/F290Y/L291M triple mutation, which also disrupts the ITIM-like motif, completely abrogated the binding of SHP-1. Interestingly, this triple mutant of IRAK-1 is identical to the corresponding site within IRAK-4. Collectively, these site-specific mutations confirm the role of the ITIM-like motif in the binding of SHP-1 to IRAK-1. This represents the first description of such a motif in a kinase that we now call KTIM (**K**inase **T**yrosyl-based **I**nhibitory **M**otif). Importantly, these experiments also suggest that the SHP-1-mediated regulation of IRAK-1 is a mechanism not shared with IRAK-4.

### *Leishmania* inhibits LPS-mediated MØ functions by rapidly inactivating IRAK-1

**Figure 4:**
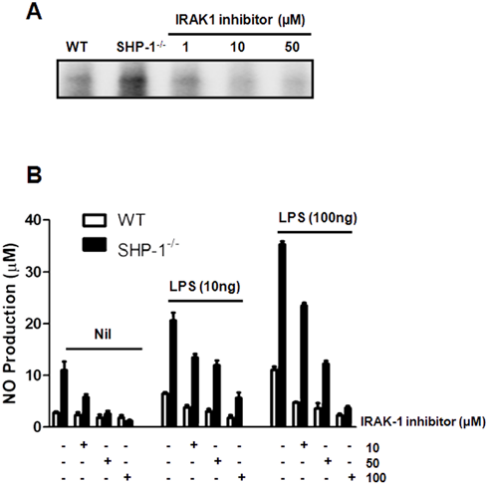
Effect of pharmacological inhibition of IRAK-1 on MØ NO production. (A) IRAK-1 was immunoprecipitated from SHP-1^−/−^ MØ lysates and incubated (1 h, RT) with increasing concentrations of the IRAK-1 inhibitor. A kinase assay was then performed to show functionality of the inhibitor. Data are representative of three experiments. (B) NO assay showing that the IRAK-1 inhibitor blocks, in a dose-dependent manner, basal production of NO by SHP-1^−/−^ cells as well as LPS-mediated (O/N stimulation) NO production in both WT and SHP-1^−/−^ MØs.

The biological relevance of this regulatory interaction between IRAK-1 and SHP-1 was investigated using the ability of N-(2-Morpholinylethyl)-2-(3-nitrobenzoylamido)-benzimidazole, a potent IRAK-1 inhibitor [19], to reduce NO production in WT and SHP-1^−/−^ MØs. As mentioned earlier, SHP-1 deficiency in MØs results in an increase in NF-κB and AP-1 activity [7]–[9] leading to NO production at basal level and in response to LPS when compared to WT [8]. Addition of the IRAK-1 inhibitor abrogated IRAK-1 activity in a dose-dependent manner (Figure 4A), and was paralleled by a reduction of basal NO production in SHP-1^−/−^ cells and in LPS-mediated NO production in both cell lines (Figure 4B). In addition to demonstrating the essential role of IRAK-1 signalling in NO generation, our data also shows that SHP-1-mediated IRAK-1 regulation is critical for the control of MØ activation.

**Figure 5:**
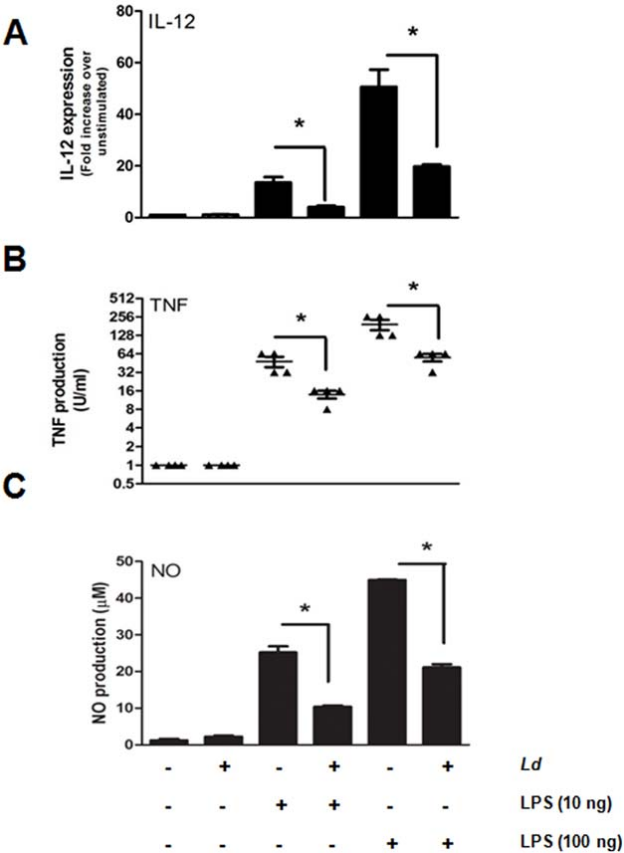
Inhibition of LPS-mediated functions by *Leishmania*. (A) LPS-mediated MØ IL-12 mRNA expression was analyzed by RT-PCR in uninfected and *Leishmania*-infected MØs. Cells were infected with *L. donovani* O/N followed by LPS stimulation (10 and 100 ng/ml, 12 h). (B) LPS-mediated TNF production by MØs infected with *Leishmania*. Cells have been infected as above and stimulated with LPS for 3 h. (C) NO production by *Leishmania*-infected MØs in response to LPS. Cells have been infected as above and stimulated with LPS for 24 h. (A–C) *, significant at *P*<0.05, Anova test, error bar SEM. Mean of three independent experiments.

Using *Leishmania* as an infectious model, we studied its ability to inhibit key MØ LPS-mediated functions namely: IL-12 expression, TNF production, and NO generation. Our results confirmed that infection with *Leishmania* caused a significant inhibition of LPS-mediated expression of IL-12 (Figure 5A), TNF production (Figure 5B), and NO generation (Figure 5C) in MØs.

**Figure 6:**
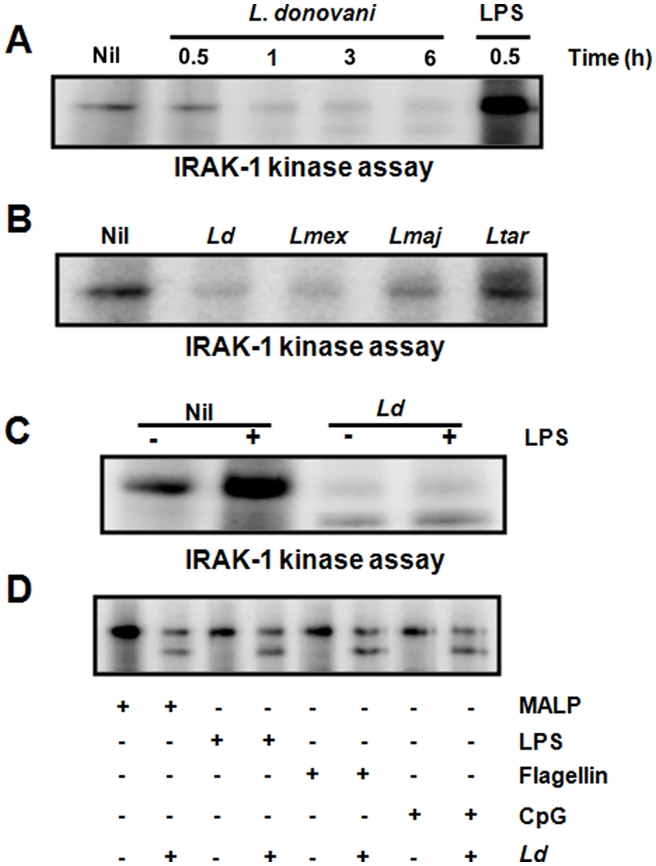
Inhibition of LPS-induced IRAK-1 kinase activity by *Leishmania*. (A) Kinase assay performed on IRAK-1 IPs from lysates of MØs uninfected and infected with *L. donovani* over a 6 h time-period. LPS stimulation (100 ng/ml, 30 min), positive control. (B) IRAK-1 kinase activity detected in IPs from lysates of MØs infected or not with pathogenic *Leishmania* species (*L. donovani, L. mexicana, L. major*) (20∶1 parasite to cell ratio, 1 h). Non-pathogenic lizard *L. tarentolae* was used as negative control. (C) Kinase assay of IRAK-1 IPs from lysates of naïve and *L. donovani*-infected MØs (O/N infection) subjected or not to LPS stimulation (100 ng/ml, 30 min). (D) IRAK-1 kinase activity in IPs from naïve and *L. donovani*-infected MØs (O/N infection) stimulated or not with various TLR ligands (MALP (100 ng/ml), LPS (100 ng/ml), flagellin (100 ng/ml), CpG (5 µg/ml); 30 min). All results are representative of at least three independent experiments.

As *Leishmania* activates host SHP-1 and blocks many LPS-mediated functions known to be detrimental to the parasite, we investigated the possibility that *Leishmania* inactivates IRAK-1. Kinase assays comparing IRAK-1 activity in MØs infected with *L. donovani* to uninfected cells revealed that the parasite caused a rapid time-dependent inactivation of IRAK-1 seen by reduced basal IRAK-1 activity in infected MØs (Figure 6A). To investigate whether IRAK-1 inactivation is a common mechanism utilized by other infectious *Leishmania* species, MØs were infected for 1 h with various *Leishmania* species promastigotes and IRAK-1 kinase activity was measured. *L. donovani* decreased IRAK-1 activity by 65±11% SD, and consistent with our expectation, *L. mexicana* and *L. major* were also able to inactivate IRAK-1 as they decreased IRAK-1 kinase activity by 65±7% SD and 52±4% SD, respectively (Figure 6B). Interestingly, *L. tarentolae*, a lizard non-pathogenic *Leishmania* did not inhibit IRAK-1 and seemed to even slightly activate it (increase of 20±11% SD).

In light of these observations, we were interested to evaluate whether the *Leishmania*-mediated IRAK-1 kinase inactivation could alter LPS-mediated functions in infected MØs. Our results indicated that unlike LPS stimulation *per se* that activates IRAK-1, infection with *Leishmania* rendered IRAK-1 activation refractory to this TLR4 agonist (Figure 6C). Since IRAK-1 signals downstream of all TLRs with the exception of TLR3, we investigated whether this *Leishmania*-induced IRAK-1 inactivation is persistent upon stimulation with other TLR ligands. As expected, *Leishmania* was able to render IRAK-1 unresponsive to MALP (TLR2), flagellin (TLR5), and CpG (TLR9) (Figure 6D). These results suggest that alteration of IRAK-1-dependent signalling by *Leishmania* causes a general unresponsiveness to a broad range of TLR ligands. All TLR ligands used were shown to be functional using an NF-κB nuclear translocation assay (Figure S3).

### *Leishmania* infection enhances the IRAK-1/SHP-1 interaction leading to IRAK-1 inactivation

**Figure 7:**
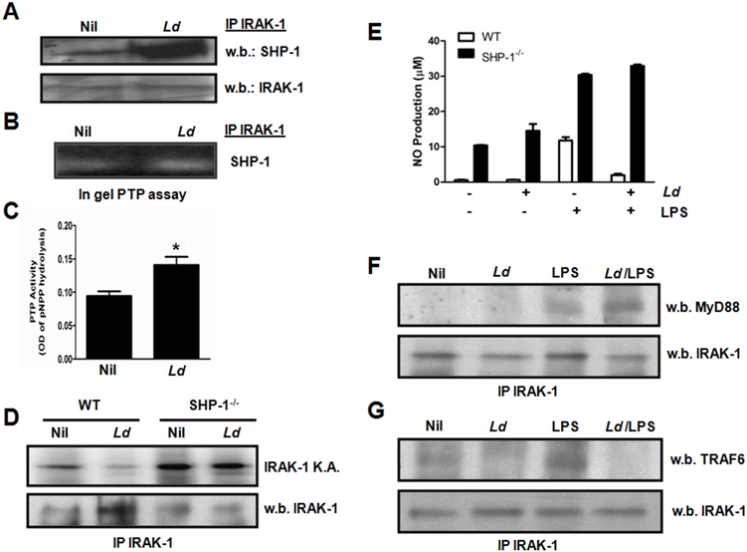
IRAK-1/SHP-1 interaction is enhanced by *Leishmania* infection and leads to IRAK-1 signalling alteration and MØ functional inhibition. (A) Western blot analysis demonstrating the enhanced co-IP of IRAK-1 and SHP-1 in response to *Leishmania* infection. IRAK-1 was immunoprecipitated from uninfected and *Leishmania*-infected MØ lysates (30 min post-infection). The IPs were run on SDS-PAGE and blotted against SHP-1 (upper panel). Membrane was then stripped and blotted against IRAK-1 as a control for equal IP (lower panel). (B) A fraction of the IPs (a, upper panel) was subjected to in gel PTP assay showing higher SHP-1 activity associated with the IP of *Leishmania*-infected MØs over uninfected. (C) Fraction of the IP (a, upper panel) was also subjected to a phosphatase assay based on pNPP hydrolysis demonstrating a significantly higher total phosphatase activity in the IP of *Leishmania*-infected cells compared to uninfected controls. *, *P*<0.05; error bar SD. Data are the mean of four independent experiments. (D) IRAK-1 kinase assay of WT and SHP-1^−/−^ MØs infected or not with *L. donovani* for 1 h (upper panel). IP fraction was kept and subjected to western blot as a loading control of IRAK-1 immunoprecipitation (lower panel). (E) NO production by *Leishmania*-infected WT and SHP-1^−/−^ MØs in response to LPS. Cells have been infected with *L. donovani* (O/N) and stimulated with LPS for 24 h. Significant difference *P*<0.05, Anova test, error bar SEM. Mean of three independent experiments. (F and G) IRAK-1 Inactivation by *Leishmania* causes its inability to bind TRAF6. Western blot analysis showing that *Leishmania* causes an abrogation of the ability of IRAK-1 to bind TRAF6, but not MyD88, upon LPS stimulation. IRAK-1 was immunoprecipitated from lysates of naïve and *L. donovani*-infected MØ (1 h infection) stimulated or not with LPS (100 ng/ml, 1 h). The IPs were run on SDS-PAGE and blotted against MyD88 (F) and TRAF6 (G). Membranes were stripped and blotted against IRAK-1 to demonstrate equal IP.

Having previously reported that *Leishmania* can rapidly induce host PTP SHP-1 to inactivate JAK and MAP kinase pathways [8],[20], we hypothesized that the *Leishmania*-induced IRAK-1 inactivation observed was associated with an increased SHP-1/IRAK-1 interaction. We indeed noticed by Western blot that a significantly greater amount of SHP-1 was co-immunoprecipitated with IRAK-1 upon *Leishmania* infection (Figure 7A). Similarly, using in gel PTP assay, we were able to detect more SHP-1 activity in IRAK-1 IP from lysates of *Leishmania*-infected MØs (Figure 7B). Higher SHP-1 activity in *Leishmania* infected MØs was further supported when equal IP fractions were subjected to a pNPP phosphatase assay (Figure 7C).

To demonstrate that the increased SHP-1/IRAK-1 binding upon *Leishmania* infection is responsible for IRAK-1 inactivation, IRAK-1 kinase activity was monitored in infected WT and SHP-1^−/−^ MØs. In accordance with our finding in B10R MØs (Figure 6), *Leishmania* was able to inactivate IRAK-1 in WT MØs (67±9% SD decrease in IRAK-1 activity). Interestingly, this *Leishmania-*induced inactivation was not detected in the absence of SHP-1 (2±6% SD decrease in IRAK-1 activity) (Figure 7D). This rescue of IRAK-1 activity was correlated with an inability of the parasite to block LPS-induced NO production in SHP-1^−/−^ MØs (Figure 7E). Collectively, this set of data shows that the *Leishmania*-activated SHP-1 is responsible for IRAK-1 inactivation leading to the unresponsiveness of infected MØs to LPS stimulation.

To further understand the impact of IRAK-1 inactivation on LPS-mediated activation in infected MØs, we monitored the association and dissociation events of IRAK-1 with known key signalling molecules (MyD88, TRAF6) in response to LPS in naïve and *Leishmania*-infected cells. The result showed that IRAK-1 inactivation by *Leishmania*-induced SHP-1 is associated with the inability of IRAK-1 to detach from MyD88 and attach to TRAF6 in response to LPS stimulation (Figures 7F and G).

### IRAK-1 and SHP-1 emerged in early vertebrates while IRAK-1 KTIM appeared only in amphibians

**Figure 8:**
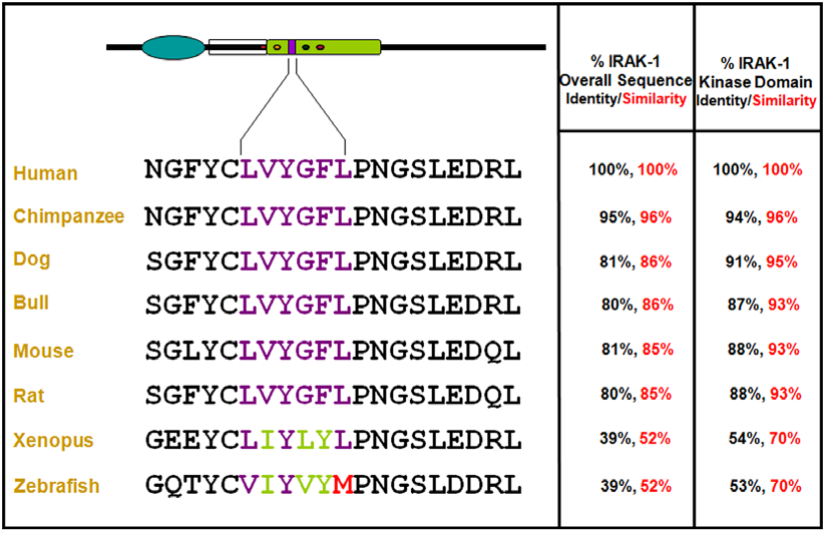
IRAK-1 KTIM is evolutionarily conserved in vertebrates. Sequence comparison of IRAK-1 in various vertebrates reveal that the KTIM is conserved in all vertebrates down to *Xenopus tropicalis* (amphibian) but is absent in *Danio rerio* (zebrafish). All homology percentages were calculated using the human IRAK-1 sequence as a reference. Human: *Homo sapiens*; Chimpanzee: *Pan troglodytes*; Dog: *Canis familiaris*; Bull: *Bos Taurus*; Mouse: *Mus musculus*; Rat: *Rattus norvegicus*.

**Figure 9:**
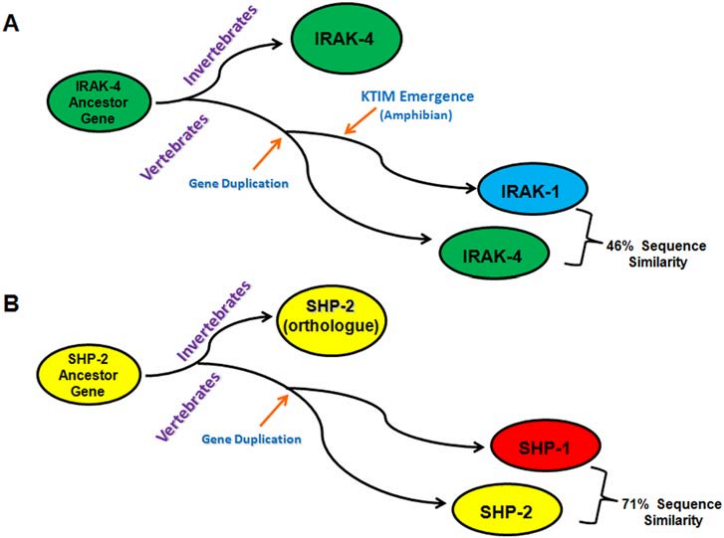
Evolution of vertebrate IRAK-1 and SHP-1 from IRAK-4 and SHP-2 ancestor genes. Schematic representation of the appearance of IRAK-1 and SHP-1 from gene duplication events of the IRAK-4 (A) and SHP-2 (B) ancestor genes, respectively. The emergence of the KTIM in IRAK-1 occurred after this gene duplication event took place as the motif only appeared in amphibians. Similarity percentages were calculated using the mouse IRAK-1 and IRAK-4 sequences.

Given the important regulatory function of the KTIM present within IRAK-1, we speculated that it would be evolutionarily conserved. *In silico* sequence comparisons of available IRAK-1 sequences revealed that KTIM (**L**V**Y**GF**L**) was fully conserved from rodents to human (Figure 8). However, while the KTIM in *Xenopus tropicalis* showed some variations compared to the other vertebrate sequences (**L** I **Y** LY **L**), it was absent in zebrafish due to the presence of a methionine at the last position (**V** I **Y** VYM). Next, we addressed the origin of IRAK-1 and SHP-1 as they are only present in vertebrates. Sequence similarity analyses, including available IRAK-4 sequences from vertebrates and invertebrates (Figure S4), indicate that IRAK-1 evolved from IRAK-4 by gene duplication (Figure 9A). Similar sequence similarity comparisons suggest that SHP-1 evolved from SHP-2 and its orthologues found in invertebrates and that the ancestral SHP-1 gene also appeared through gene duplication in lower vertebrates (zebrafish) (Figure 9B).

**Figure 10:**
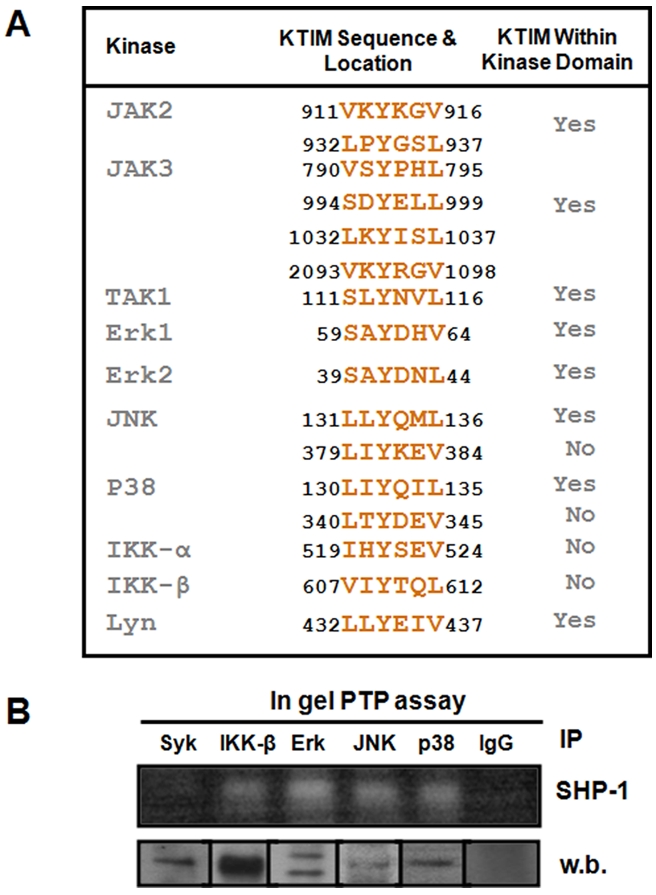
Several kinases possess KTIMs. (A) Table showing that several kinases from the JAK, MAP, Src, and IKK kinase families possess potential KTIMs in their amino acid sequences. Screening was done using published mouse protein sequences found in the NCBI protein database. (B) An in gel phosphatase assay (upper panel) demonstrating that IPs of IKK-β, Erk1/2, JNK, and p38 all exhibit SHP-1 activity. Syk IP was added as a control for a kinase that has no KTIM in its sequence and rabbit IgG was used as a negative control. Fractions of all IPs were kept and run on SDS-PAGE and blotted against their corresponding antibody to demonstrate the success of the IP procedure (lower panel).

From these observations, we raised the question whether other kinases may also have a KTIM within their kinase domain. Although several proteins involved in MyD88-dependent signalling (e.g. MyD88, TIRAP, TRAF6) did not contain KTIMs in their amino acid sequence (data not shown), we were intrigued to discover that several kinases from the JAK, MAP, Src, and IKK kinase families (JAK2, JAK3, Erk1/2, JNK, p38, Lyn, IKKα/β) contained one or more potential KTIMs, the majority located within their kinase domains (Figure 10A). This finding raises the possibility that KTIMs play important regulatory functions for many kinases by favoring their interaction with SHP-1, as we herein report for IRAK-1. SHP-1 binding may control the activity of these kinases at resting state or regulate their activity upon activation. In gel phosphatase assays that we performed support this possibility as they demonstrate that IPs of IKK-β, Erk, JNK, and p38 indeed exhibit SHP-1 activity (Figure 10B), indicating that these kinases interact with SHP-1. Interestingly, Syk – a kinase that has no KTIM in its amino acid sequence – did not show interaction with SHP-1 at resting state.

## Discussion

*Leishmania* has been reported to inhibit critical LPS-mediated MØ functions such as NO and pro-inflammatory cytokines (e.g. IL-12 and TNF) production [2],[4],[5]. Although mechanisms whereby NO is inhibited by *Leishmania* in response to IFN-γ have been well explored [2], our knowledge concerning the negative regulatory mechanisms leading to the down-regulation of LPS-mediated MØ functions in *Leishmania*-infected cells is limited. Herein, we provide the first demonstration that the *Leishmania* parasite can rapidly inactivate IRAK-1 kinase activity with the participation of SHP-1, therefore inhibiting MØ LPS-mediated functions. We further reveal that the mechanism by which this inactivation occurs is through the binding of SHP-1 to an evolutionarily-conserved ITIM-like motif located in the kinase domain of IRAK-1. This is the first demonstration that a pathogen can use a host PTP to inactivate IRAK-1 and therefore block signalling pathways ultimately leading to free radicals and pro-inflammatory cytokines production known to be detrimental to its survival.

Given that TNF is a potent MØ activator, NO is leishmanicidal, and IL-12 is a critical cytokine that drives Th1 responses essential for the development of immunity against *Leishmania*, it is not surprising that the parasite has evolved means to block the production of these molecules [2]. A role for *Leishmania* phosphoglycans (PG) has been proposed in the inhibition of NO [21]. In addition, roles for promastigote PG [22],[23] and amastigote cysteine peptidases [24] in the inhibition of LPS-mediated IL-12 production have been reported. Nevertheless, apart from very few reports about *Leishmania*-induced alterations in the Erk MAPK [22] and the downstream transcription factor NF-κB [24], very little is known about how LPS-mediated functions are inhibited by *Leishmania*. In this study, we confirmed that all three LPS-mediated MØ functions were inhibited by *Leishmania*. Importantly, looking at NO production as a key function involved in the killing of *Leishmania* parasites, we were able to show that IRAK-1 signalling is key for its production. In fact, our finding that *Leishmania* inactivates IRAK-1 kinase activity and that this inactivation is persistent upon subsequent LPS-stimulation supports the fact that the parasite is able to successfully block LPS-mediated NO production in MØs. Interestingly, consistent with the fact that IRAK-1 signals downstream of many TLRs, we showed that IRAK-1 was also unresponsive in *Leishmania*–infected cells subjected to stimulation with TLR2, TLR5, and TLR9 ligands. This result suggests that the parasite causes wide range unresponsiveness to TLR signalling upon infection possibly allowing *Leishmania* to avoid any harmful MØ activation involving TLR engagement. Interestingly, *L*. *donovani* has been shown to activate IRAK-1 in IFN-γ-primed MØs [25] suggesting that the activation state of the MØ can play an important role in the ability of the parasite to inactivate IRAK-1. In an effort to understand how *Leishmania* inactivates IRAK-1, we were able to identify SHP-1 as a key player in this process as there was almost a complete rescue of IRAK-1 activity in SHP-1^−/−^ MØs infected with *Leishmania*. This rescue was corroborated by the parasite's inability to block LPS-mediated NO production in SHP-1^−/−^ MØs. These results suggest a new evasion mechanism whereby *Leishmania* can avoid detrimental MØ functions driven by MyD88-dependent pathways by blocking IRAK-1, a key kinase in this pathway.

Our observation that the *Leishmania*–mediated IRAK-1 inactivation was associated with enhanced SHP-1 binding to IRAK-1 fits with our finding that SHP-1 binds to and regulates IRAK-1 at resting state. We clearly showed that IRAK-1's intrinsic kinase activity was higher in SHP-1^−/−^ compared to WT MØs identifying SHP-1 as a novel regulator of IRAK-1 activity, a finding supported by recent work of Cao and colleagues [11]. The fact that SHP-1 interacts with and also dephosphorylates tyrosyl residues raised the possibility that IRAK-1 is tyrosine phosphorylated. Here, we show that IRAK-1 is indeed tyrosine phosphorylated at resting state, and further so in response to LPS stimulation. Given that IRAK-1 was previously shown to be phosphorylated on Ser/Thr residues only [18], our findings represent the first demonstration that IRAK-1 is also tyrosine phosphorylated.

Having identified an ITIM-like motif in the kinase domain of IRAK-1 as the binding site of SHP-1, its functionality was demonstrated by generating mutations within the motif providing valuable information about the role of its amino acid components in the binding affinity of SHP-1. Firstly, the tyrosine to phenylalanine (Y288F) mutation suggested that the phosphorylation of the motif's central tyrosine is not necessary for the binding of SHP-1 to occur. Indeed it has been previously reported that tyrosyl phosphorylation within ITIMs is not always required for the binding of SH2-domain containing proteins [26]. Secondly, the observation that the G289A-F290Y-L291M mutation caused a total abrogation of SHP-1 binding, and that the L291M mutation partially reduced binding suggested that the amino acids between the central tyrosine and the terminal leucine in the motif play an important role in the binding affinity of SHP-1. Lastly, as the triple mutant was designed to render the ITIM-like site in IRAK-1 identical to its corresponding site in IRAK-4, the loss of SHP-1 binding in this mutant suggested that the SHP-1-mediated regulation of IRAK-1 is a regulatory mechanism not shared with IRAK-4. It is noteworthy to emphasize that ITIMs have been named so due to their presence in intracytoplasmic portions of transmembrane receptors [27]. Given that here we describe this motif to be found in a cytosolic kinase and show that it mediates SHP-1 binding and IRAK-1negative regulation, we propose to rename it KTIM (**K**inase **T**yrosyl-based **I**nhibitory **M**otif).

In MyD88-dependent signalling pathways, binding of TLR ligand to its corresponding receptor causes a rearrangement of the receptor complex and triggers the recruitment of the adaptor protein MyD88, which in turn recruits the kinases IRAK-4 and IRAK-1 to the receptor complex [1]. Upon critical phosphorylations of IRAK-1 by IRAK-4 [18], IRAK-1 is partially activated and is able to get fully activated by autophosphorylation. This autophosphorylation causes IRAK-1 to detach from the MyD88 complex and attach to TRAF6 activating downstream signalling pathways. Therefore, the IRAK-1 inactivation by *Leishmania*-induced SHP-1 had to interfere somehow with the integrity of the previous signalling events. Of utmost interest, we have been able to show that although IRAK-1 was still able to bind MyD88 in *Leishmania*-infected MØs in response to LPS stimulation, the kinase was unable to detach from the MyD88 complex and bind to TRAF6 as the stimulation persisted. This is the first demonstration that a pathogen can interfere with Toll signalling by altering IRAK-1's capacity to dissociate from the MyD88 complex. This inability of IRAK-1 to detach from MyD88 is supported by our observation that the binding of *Leishmania-*induced SHP-1 to the kinase domain of IRAK-1 causes a strong inactivation of this kinase seen by its inability to autophosphorylate, a process required for IRAK-1 to detach from the receptor complex and activate downstream signalling cascades.

Finally, it was remarkable to find out that the KTIM in IRAK-1 was evolutionarily conserved from human to rodents. The absence of KTIM in fish and its appearance in amphibians suggests that this motif emerged rapidly after the appearance of the ancestral IRAK-1 gene in early branching vertebrates (amphibians) and was highly conserved thereafter (Figure S5). Our findings thus raise the possibility that during the course of evolution, the emergence of a mechanism to regulate the innate immune response by targeting IRAK-1 activity (e.g. SHP-1) may have favoured the development of a more sophisticated adaptive immune system in higher vertebrates (Figure S5). In addition, it is important to note that Toll-Interacting Protein (TOLLIP) [28], the only other negative regulator of IRAK-1 in the resting state has emerged very early in invertebrates as opposed to SHP-1 [29], IRAK-1 [30] and the KTIM motif which all appeared only in early vertebrates. Noteworthy, we found that several other kinases from the JAK, MAP and IKK kinase families contained one or more potential KTIMs raising the possibility that KTIMs play important regulatory functions in many kinases (other than IRAK-1) by favouring their interaction with SHP-1. In fact, it has been previously reported that some of these kinases (e.g. JAK2, JAK3, JNK, Erk1/2) are negatively regulated by SHP-1 [31]–[33]. However, none of these studies paid great attention to the mechanism whereby SHP-1 either interacts or regulates these kinases. It remains to be mentioned that whereas some of these kinases are also present in invertebrates, IRAK-1 and its KTIM only appeared in vertebrates. This observation supports the idea that the appearance of this motif in IRAK-1 has favoured the development of a mechanism to control the innate immune response. In this context, regulation of IRAK-1 kinase activity would have prevented abnormal and exacerbated microbicidal and inflammatory immune responses that could have been detrimental to vertebrates and to the development of the adaptive immune response. It is also tempting to speculate that the appearance of an improved control over kinases by SHP-1 may have influenced the global development of vertebrates, as several of these kinases play pivotal roles in the regulation of cellular, molecular, developmental, and metabolic processes.

In conclusion, we have identified a new evasion mechanism whereby *Leishmania*-activated SHP-1 binds to an evolutionarily conserved KTIM located in IRAK-1's kinase domain leading to its inactivation. This abrogation was associated with the inability of IRAK-1 to detach from the MyD88 complex to bind TRAF6, consequently resulting in the unresponsiveness of *Leishmania*-infected macrophages to several TLR ligand stimulation including LPS. By doing so, the parasite is not only able to block LPS-mediated MØ production of NO and pro-inflammatory cytokines known to be involved in *Leishmania* killing, but also terminate the extremely important roles played by these molecules in the development of an effective adaptive immune response. At the evolutionary level, we propose that the appearance of SHP-1 as a key regulator of IRAK-1 kinase activity represented a pivotal evolutionary step that could have favoured the development of the adaptive immune response in vertebrates.

## Supporting Information

Figure S1 IRAK-1 contains a KTIM motif in its kinase domain. The full amino acid sequence of mouse IRAK-1 has been obtained from the NCBI protein database (Ref. no. Q62406). The newly identified KTIM is in violet. Bottom drawing is a schematic representation of the IRAK-1 protein showing the locations of the different domains and critical residues. KTIM motif is shown as a violet rectangle. ProST, Proline/Serine/Threonine -rich.
(1.56 MB TIF)

Figure S2 Among the IRAK family, KTIM is unique to IRAK-1. All other IRAK family members (IRAK-2, IRAK-M, and IRAK-4) whose sequences are available for various invertebrate and vertebrate organisms lack a KTIM. A sequence comparison in the KTIM region among the different IRAK family members is shown. Mouse was chosen as a representative organism.
(0.38 MB TIF)

Figure S3 TLR ligands activate NF-κB in stimulated MØs. Gel represents an electromobility shift assay (EMSA) showing NF-κB nuclear translocation in response to a 2 h stimulation with the different TLR ligands used in Figure 6D. The EMSA confirms that the ligands are functional and activating at the concentrations used. MALP, Macrophage-activating lipopeptide-2. Flag, Flagellin. S, Specific competition (100× cold oligo). NS, Non-specific competition (SP1 oligo).
(0.19 MB TIF)

Figure S4 IRAK-4 shows homology to IRAK-1 but does not bear a KTIM due to a single amino acid substitution. IRAK-4 sequence comparison of various vertebrates and invertebrates reveal that IRAK-4 has no KTIM due to a single leucine to methionine/isoleucine substitution. All IRAK-4 homology percentages were calculated using the human IRAK-4 sequence as a reference. IRAK-1/IRAK-4 homology percentages were calculated within the same species. Rhesus monkey: *Macaca mulatta*; Chicken: *Gallus gallus*; Squid: *Euprymna scolopes*; Sea urchin: *Strongylocentrotus purpuratus*; Worm: *Caenorhabditis elegans*; Honeybee: *Apis mellifera*; Fly: *Drosophila melanogaster*.
(1.72 MB TIF)

Figure S5 Regulation of IRAK-1 by SHP-1 through its binding to KTIM is unique to vertebrates and may have favoured the development of their adaptive immune response. Schematic representation of the emergence of IRAK-1 and SHP-1 from IRAK-4 and SHP-2, respectively. Unlike TOLLIP and SHP-2 which are found in invertebrates, SHP-1 arose in vertebrates just like IRAK-1 and KTIM, coinciding with the emergence of the adaptive immune response.
(1.54 MB TIF)

Alternative Language Abstract S1 Translation of the Abstract into Arabic by Issa Ayoub Abu-Dayyeh
(0.02 MB DOC)

Alternative Language Abstract S2 Translation of the Abstract into French by Marceline Côté
(0.02 MB DOC)

Alternative Language Abstract S3 Translation of the Abstract into Spanish by Maria-Adelaida Gomez and Irazu Contreras
(0.02 MB DOC)

Alternative Language Abstract S4 Translation of the Abstract into Farsi (Persian) by Kasra Hassani
(0.02 MB DOC)
