# Supplementary figures and images for: Leishmania-Induced IRAK-1 Inactivation Is Mediated by SHP-1 Interacting with an Evolutionarily Conserved KTIM Motif
Source: PLoS Negl Trop Dis. 2008 Dec 23;2(12):e305. doi: 10.1371/journal.pntd.0000305 (PMC2596967; doi:10.1371/journal.pntd.0000305)

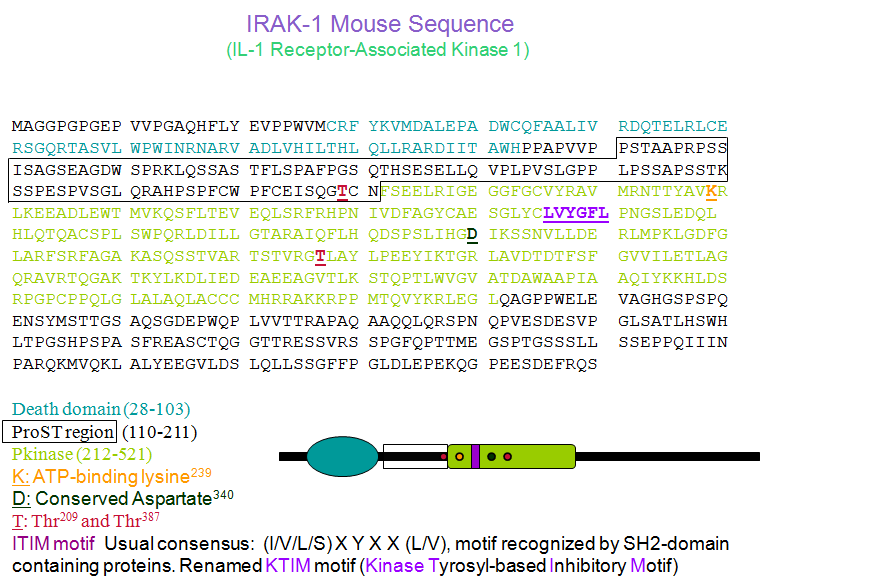

Supplement: Figure S1 — IRAK-1 contains a KTIM motif in its kinase domain. The full amino acid sequence of mouse IRAK-1 has been obtained from the NCBI protein database (Ref. no. Q62406). The newly identified KTIM is in violet. Bottom drawing is a schematic representation of the IRAK-1 protein showing the locations of the different domains and critical residues. KTIM motif is shown as a violet rectangle. ProST, Proline/Serine/Threonine -rich. (1.56 MB TIF) [file pntd.0000305.s001.tif]

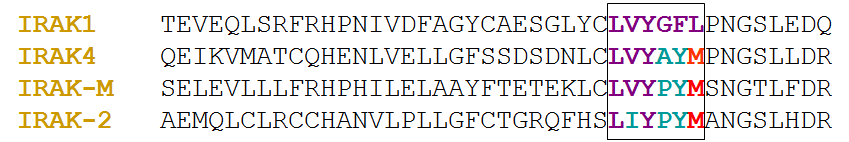

Supplement: Figure S2 — Among the IRAK family, KTIM is unique to IRAK-1. All other IRAK family members (IRAK-2, IRAK-M, and IRAK-4) whose sequences are available for various invertebrate and vertebrate organisms lack a KTIM. A sequence comparison in the KTIM region among the different IRAK family members is shown. Mouse was chosen as a representative organism. (0.38 MB TIF) [file pntd.0000305.s002.tif]

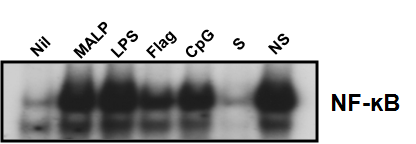

Supplement: Figure S3 — TLR ligands activate NF-κB in stimulated MØs. Gel represents an electromobility shift assay (EMSA) showing NF-κB nuclear translocation in response to a 2 h stimulation with the different TLR ligands used in Figure 6D. The EMSA confirms that the ligands are functional and activating at the concentrations used. MALP, Macrophage-activating lipopeptide-2. Flag, Flagellin. S, Specific competition (100× cold oligo). NS, Non-specific competition (SP1 oligo). (0.19 MB TIF) [file pntd.0000305.s003.tif]

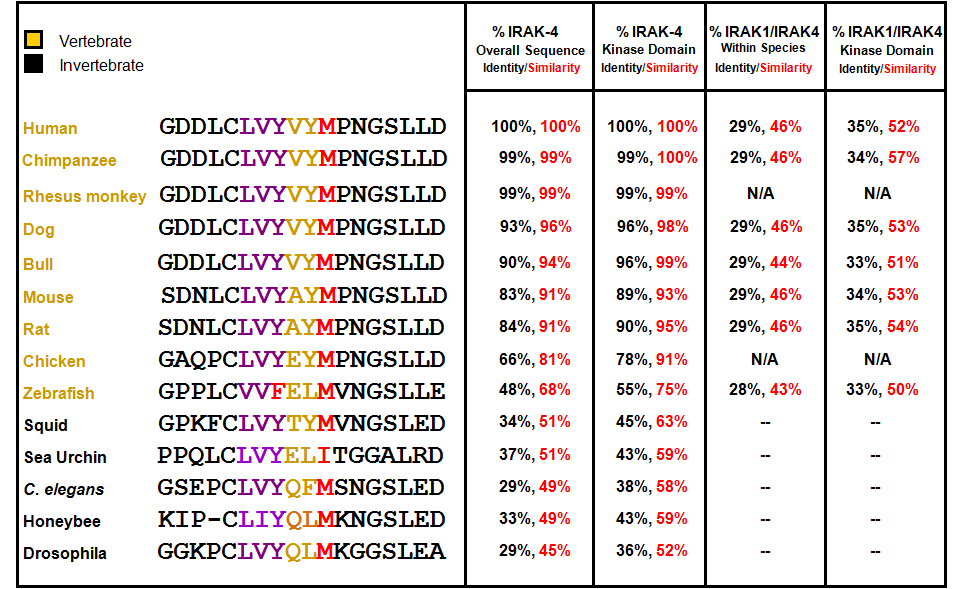

Supplement: Figure S4 — IRAK-4 shows homology to IRAK-1 but does not bear a KTIM due to a single amino acid substitution. IRAK-4 sequence comparison of various vertebrates and invertebrates reveal that IRAK-4 has no KTIM due to a single leucine to methionine/isoleucine substitution. All IRAK-4 homology percentages were calculated using the human IRAK-4 sequence as a reference. IRAK-1/IRAK-4 homology percentages were calculated within the same species. Rhesus monkey: Macaca mulatta; Chicken: Gallus gallus; Squid: Euprymna scolopes; Sea urchin: Strongylocentrotus purpuratus; Worm: Caenorhabditis elegans; Honeybee: Apis mellifera; Fly: Drosophila melanogaster. (1.72 MB TIF) [file pntd.0000305.s004.tif]

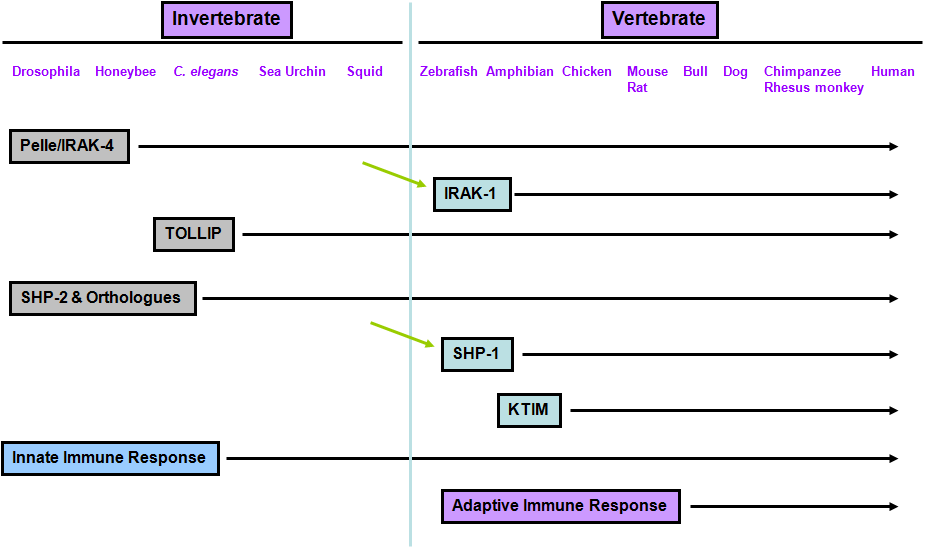

Supplement: Figure S5 — Regulation of IRAK-1 by SHP-1 through its binding to KTIM is unique to vertebrates and may have favoured the development of their adaptive immune response. Schematic representation of the emergence of IRAK-1 and SHP-1 from IRAK-4 and SHP-2, respectively. Unlike TOLLIP and SHP-2 which are found in invertebrates, SHP-1 arose in vertebrates just like IRAK-1 and KTIM, coinciding with the emergence of the adaptive immune response. (1.54 MB TIF) [file pntd.0000305.s005.tif]
