# Supplementary material for: Leishmania-Induced IRAK-1 Inactivation Is Mediated by SHP-1 Interacting with an Evolutionarily Conserved KTIM Motif
Source: PLoS Negl Trop Dis. 2008 Dec 23;2(12):e305. doi: 10.1371/journal.pntd.0000305 (PMC2596967; doi:10.1371/journal.pntd.0000305)
Supplement: Alternative Language Abstract S1 — Translation of the Abstract into Arabic by Issa Ayoub Abu-Dayyeh (0.02 MB DOC) [file pntd.0000305.s006.doc]

Arabic translation of the abstract provided by**: Issa Ayoub Abu-Dayyeh**

**الکائن الطُّفيلي المعروف باللِّیشمانيا قادر علی العيش و التَّکاثر داخل خلايا البلعمة (Phagocytes) بسلام مستخدماً أساليب متعددة للحدِّ من قدرات هذه الخلايا في القضاء علی مسببات الأمراض. لقد نشرنا في السابق أبحاثاً تفيد بأنَّ الليشمانيا فضلاً عن LPS البکتيري يؤديان إلی نشوء إلتهاب قوي في الفئران و الخلايا التي تفتقر إلى الفسفتاز SHP-1. هذه الملاحظة جعلتنا نبحث في الدور المحتمل ﻟِ SHP-1 في تنظيم سير انتقال الرسالة (Cell Signalling) الناتجة عن Toll-Like Receptors (TLRs) و بالأخص تفاعل SHP-1 مع أحد الکاينزات الأساسية في سلسلة انتقال رسالة TLR ألا و هو: IRAK-1.**

**في هذا البحث، تمًّ اکتشاف طريقة جديدة تتَّبعها الليشمانيا لوقف سير انتقال رسالة TLR في خلايا البلعمة و ذﻟﻚ من خلال قيامها بتنشيط عمل SHP-1 والذي بدوره يقوم بتعطيل IRAK-1 مانعاً إنتاج أکسيد النترﻳﻚ و مواد أخری ضرورية للقضاء علی الجراثيم التي تدخل الجسم.**

**لقد تمکَّنا أيضاً من تحديد موقع التفاعل بين الفوسفتاز SHP-1 و الکايناز IRAK-1 باستخدام الطَّفرات الجينية. کما وجدنا بأن موقع التَّفاعل هو موتيف (Motif) يتکوَّن من ستَّة أحماض أمينية محفوظة تماماً في عملية التَّطور الطبيعي من مرحلة البرمائيَّات إلی الإنسان الحديث و أنَّ مثل هذا الموقع موجود أيضاً في کاينازات أخری في الخلية (مثل Erk 1/2 و IKK α/β) و بالتَّالي يمثِّل موتيف يمکن له أن يؤدي دوراً مهمّاً في تنظيم عمل ﺗﻠﻚ البروتينات. و من هنا تأتي تسميتنا له ﺑِ KTIM.**

**بناءً علی النتائج السابقة، فإنَّ هذا البحث يمثِّل أول عرض تجريبي علی قدرة جرثومة علی استخدام فوسفتاز خلوي (SHP-1) للحيلولة دون عمل کيناز (IRAK-1) في محاولةٍ لتعطيل عمل خلية البلعمة و إحباط سعيها صوب القضاء عليها.**
