# Supplementary material for: Leishmania-Induced IRAK-1 Inactivation Is Mediated by SHP-1 Interacting with an Evolutionarily Conserved KTIM Motif
Source: PLoS Negl Trop Dis. 2008 Dec 23;2(12):e305. doi: 10.1371/journal.pntd.0000305 (PMC2596967; doi:10.1371/journal.pntd.0000305)
Supplement: Alternative Language Abstract S2 — Translation of the Abstract into French by Marceline Côté (0.02 MB DOC) [file pntd.0000305.s007.doc]

French translation of the abstract provided by: **Marceline Côté**

**Afin de favoriser leur survie et leur prolifération, les parasites du genre *Leishmania* peuvent altérer rapidement plusieurs voies de signalisation chez les macrophages infectés résultant ainsi en l’atténuation de l’inflammation et de la réponse immunitaire immédiate. Suite à notre récente étude démontrant que *Leishmania* et le LPS bactérien entraînent une hausse significative de la réponse immunitaire chez les animaux et les phagocytes déficients en la protéine tyrosine phosphatase (PTP) SHP-1, nous avons émis l’hypothèse que *Leishmania* utilise SHP-1 afin d’inactiver des kinases-clées impliquées dans l’immunité innée et les cascades de signalisation des *Toll-Like Receptors* (TLR) telles que l’*IL-1 Receptor-Associated Kinase 1* (IRAK-1). Dans la présente étude, nous démontrons que, lors de l’infection par *Leishmania*, SHP-1 interagit avec IRAK-1 causant l’inactivation complète de l’activité kinase de cette dernière et, par conséquent, inhibe une activation subséquente par le LPS et abroge les fonctions normales du macrophage. De plus, nous avons découvert que SHP-1 lie IRAK-1 via un motif ressemblant à un ITIM mais se trouvant dans le domaine kinase d’IRAK-1 que nous avons conséquemment nommé KTIM (*Kinase Tyrosyl-Based Inhibitory Motif).* Ce motif est apparu tôt chez les vertébrés et est absent de tous les autres membres de la famille IRAK. Nos résultats révèlent aussi que plusieurs kinases telles que Erk1/2 et IKK-α/β impliquées dans les voies signalétiques des TLR contiennent également des KTIMs dans leur domaine kinase et peuvent interagir avec SHP-1. Ainsi, il s’agit de la première démonstration qu’un pathogène possède la capacité de moduler SHP-1, une PTP de la cellule hôte, afin d’inactiver directement IRAK-1 via un motif KTIM qui a été conservé au cours de l’évolution.**
