# Supplementary material for: Leishmania-Induced IRAK-1 Inactivation Is Mediated by SHP-1 Interacting with an Evolutionarily Conserved KTIM Motif
Source: PLoS Negl Trop Dis. 2008 Dec 23;2(12):e305. doi: 10.1371/journal.pntd.0000305 (PMC2596967; doi:10.1371/journal.pntd.0000305)
Supplement: Alternative Language Abstract S3 — Translation of the Abstract into Spanish by Maria-Adelaida Gomez and Irazu Contreras (0.02 MB DOC) [file pntd.0000305.s008.doc]

Spanish translation of the abstract provided by: **Maria-Adelaida Gomez and Irazú Contreras.**

**Los parásitos protozoarios del genero *Leishmania* alteran rápidamente múltiples vías de señalización del macrófago (MΦ) con el fin de inactivar la respuesta inmune innata y la inflamacion, promoviendo de esta forma su supervivencia y propagación en el huésped. Los Animales y MΦs deficientes en la fosfatasa de protein-tirosina (PTP) SHP-1 muestran un marcado aumento en la respuesta inflamatoria hacia *Leishmania* y el lipopolisacárido (LPS) bacteriano. De acuerdo a estas observaciones, postulamos que *Leishmania* utiliza SHP-1 para inactivar cinasas claves involucradas en las vías de señalización Toll-Like-Receptor (TLR) y IL-1 receptor-associated kinase 1 (IRAK-1). En este artículo reportamos la rápida interacción de SHP-1 e IRAK-1 en respuesta a la infección, la cual inactiva completamente la actividad intrínseca de la cinasa, su subsecuente activación dependiente de LPS, así como las funciones del MΦ. Así mismo, demostramos que la interacción SHP-1/IRAK-1 esta mediada por un motivo presente en el dominio activo de IRAK-1, similar a los motivos ITIM, el cual hemos denotado como KTIM (Kinase Tyrosyl-based Inhibitory Motif). A través de la evolución, este motivo regulador aparece en vertebrados tempranos y no se encuentra en ningún otro miembro proteico de la familia IRAK. Adicionalmente, nuestro estudio muestra adicionalmente que otras cinasas (Erk1/2, IKKα/β) involucradas en la regulacion de la señalizacion TLR contienen el motivo KTIM, e interactúan con SHP-1. Por lo tanto, presentamos la primera demostración en la que un patógeno utiliza una fosfatasa del huésped (SHP-1) para inactivar directamente IRAK-1 a través del motivo conservado KTIM.**
