# Supplementary material for: Leishmania-Induced IRAK-1 Inactivation Is Mediated by SHP-1 Interacting with an Evolutionarily Conserved KTIM Motif
Source: PLoS Negl Trop Dis. 2008 Dec 23;2(12):e305. doi: 10.1371/journal.pntd.0000305 (PMC2596967; doi:10.1371/journal.pntd.0000305)
Supplement: Alternative Language Abstract S4 — Translation of the Abstract into Farsi (Persian) by Kasra Hassani (0.02 MB DOC) [file pntd.0000305.s009.doc]

Farsi (Persian) translation of abstract provided by**: Kasra Hassani**

**انگل لشمانيا قادر است بسياری از مسيرهای انتقال پيام در ماکروفاژها را به سود خود دستکاری کند و بدين ترتيب التهاب و پاسخ ايمنی ذاتی را مهار کرده و در درون ماکروفاژ به رشد و تکثير بپردازد. ما پيشتر نشان داديم که لشمانيا و LPS در سلول ها و حيواناتی که فاقد تيروزين فسفاتاز SHP-1 هستند پاسخ التهابی بسيار شديدتری را القا می کنند. در نتيجه ما بر آن شديم تا ببينيم که آيا لشمانيا قادر است که از SHP-1 بهره گيرد تا کينازهای کليدی در مسير انتقال پيام Toll-Like-Receptorها نظير IRAK-1 را مهار کند يا خير. در اينجا ما نشان داده ايم که بلافاصله پس از عفونت ماکروفاژ با انگل, SHP-1 به IRAK-1 متصل می شود و به کلی تمامی فعاليت کينازی IRAK-1 و در نتيجه ی واکنش های ماکروفاژ را مهار می کند. ما همچنين نشان داده ايم که برهمکنش بين SHP-1 و IRAK-1 از طريق يک موتيف حفظ شده رخ می دهد. اين موتيف تنظيمی در مهره داران اوليه پديد آمده و ديگر اعضای خانواده ی IRAK وجود ندارد. مطالعات ما نشان داده اند که برخی ديگر از کينازهای سلولی نيز دارای اين موتيف هستند. به اين ترتيب, اين نخستين گزارش از توانايي يک پاتوژن در بهره گرفتن از يک تيروزين فسفاتاز (SHP-1) برای مهار مستقيم IRAK-1 از راه يک موتيف حفظ شده است.**
